# Nanostructured Oxygen Sensor - Using Micelles to Incorporate a Hydrophobic Platinum Porphyrin

**DOI:** 10.1371/journal.pone.0033390

**Published:** 2012-03-22

**Authors:** Fengyu Su, Ruhaniyah Alam, Qian Mei, Yanqing Tian, Cody Youngbull, Roger H. Johnson, Deirdre R. Meldrum

**Affiliations:** Center for Biosignatures Discovery Automation, Biodesign Institute, Arizona State University, Tempe, Arizona, United States of America; Clarkson University, United States of America

## Abstract

Hydrophobic platinum(II)-5,10,15,20-tetrakis-(2,3,4,5,6-pentafluorophenyl)-porphyrin (PtTFPP) was physically incorporated into micelles formed from poly(*ε*-caprolactone)-*block*-poly(ethylene glycol) to enable the application of PtTFPP in aqueous solution. Micelles were characterized using dynamic light scattering (DLS) and atomic force microscopy (AFM) to show an average diameter of about 140 nm. PtTFPP showed higher quantum efficiency in micellar solution than in tetrahydrofuran (THF) and dichloromethane (CH_2_Cl_2_). PtTFPP in micelles also exhibited higher photostability than that of PtTFPP suspended in water. PtTFPP in micelles exhibited good oxygen sensitivity and response time. This study provided an efficient approach to enable the application of hydrophobic oxygen sensors in a biological environment.

## Introduction

Dissolved oxygen concentrations in live cells and cellular environments are critical for many physiological and pathological processes including metabolism, cell respiration/oxygen consumption, and tissue hypoxia [1]–[10]. Fluorescence based optical oxygen sensors, such as platinum (II) porphyrins, palladium (II) porphyrins, and ruthenium polypyridyl complexes [11]–[23] were developed for measuring oxygen concentrations at cellular levels because these optical sensors: (i) are noninvasive; (ii) can be used in not only large, but also in small volumes down to a few pico-liters at the single cell level; and (iii) can be integrated with other optical sensors for simultaneously monitoring multi-parameters. These characteristics endow many advantages of the optical approach for oxygen concentration measurement, with which the popularly applied electrochemistry based oxygen sensing method can hardly compete [24]. Some optical oxygen sensing materials and membranes were successfully developed to extracellularly monitor oxygen consumption not only at the microscale, but also with high throughput, as has been demonstrated using bacteria, isolated mitochondria, cellular lines and small organisms [2], [5], [6], [8], [9], [10]. We have developed new oxygen sensors for extracellular oxygen sensing [25], [26], [27] and applied these sensors for detecting single cell oxygen consumption [7], [8]. Although many oxygen sensors have been reported, there are only a few materials that can be used for intracellular oxygen sensing, as this requires that the sensors be soluble or able to disperse well in water, exhibit good oxygen responses, and have excellent photostability. Water soluble ruthenium tris(diphenyl phenanthroline) (RuDPP) [28] and ruthenium tris(2,2′-dipyridyl)dichloride hexahydrate (RTDP) [29] were used to study intracellular oxygen concentrations. Polymer nanoparticles incorporated with oxygen sensitive fluorophores, such as the RuDPP and platinum (II) octaethyl porphyrin ketone (PtOEP), were prepared and loaded into cells through microinjection or microprojectile delivery [13], [30]. Liposome was utilized to deliver the RuDPP dispersed in polystyrene beads into macrophages through phagocytosis [31]. Hydrophilic metal porphyrins (PtCPK, PtCP, PdCPK, PtTCPP) [32], [33], [34] were recently studied as molecular intracellular oxygen sensors. However, extremely low quantum efficiencies, in the range of 0.001 to 0.0095, were observed for these hydrophilic metal-porphyrin-derived oxygen sensors.

**Figure 1:**
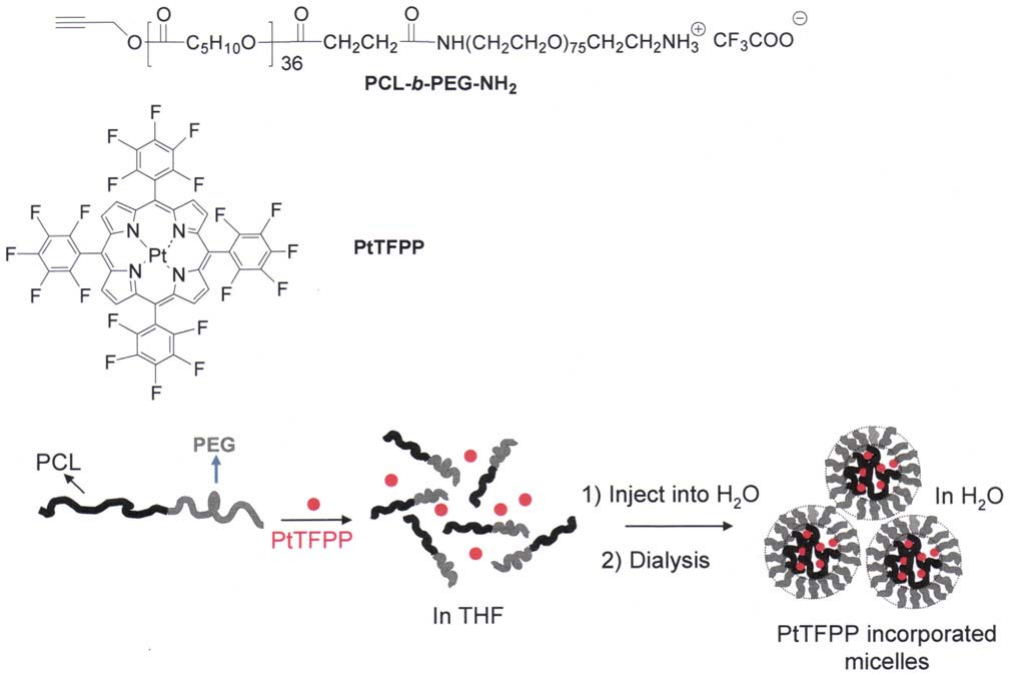
Chemical structures of PtTFPP and PCL-*b*-PEG and the schematic drawing of the micelle formation.

Among the many oxygen sensors, platinum(II)-5,10,15,20-tetrakis-(2,3,4,5,6-pentafluorophenyl)-porphyrin (PtTFPP) is known to be an excellent one due to its good responses to oxygen concentrations, high photostability, and high quantum efficiency as compared with others [35], [36]. Unfortunately, its extreme hydrophobicity limits its application in a biological environment. Recently, Borisov et al. reported an incorporation of PtTFPP in polystyrene-*block*-polyvinylpyrrolidone beads and studied the oxygen responses in aqueous solutions [37]. The authors demonstrated that the beads with an average diameter of approximately 250 nm were cell impermeable for *E. coli*. Previously, we used micelles formed from amphiphilic block copolymers to incorporate hydrophobic two-photon absorbing materials and photodynamic therapeutic drugs – porphyrins to deliver the hydrophobic materials into cells [38], [39], [40]. Along this line, herein, we report the incorporation of PtTFPP in micelles formed from poly(ethylene glycol)-*block*-poly(*ε*-caprolactone) (PEG-*b*-PCL) (Figure 1) and the evaluation of oxygen responses of PtTFPP-encapsulated micelles. We demonstrated that PtTFPP in micelles showed good oxygen responses and photo-stability in aqueous media. This study may provide an alternative avenue for intracellular oxygen sensor design and investigation.

## Experiment

### Materials

PtTFPP was purchased from Frontier Scientific (Logan Utah), PEG-*b*-PCL [*M*_n_ (GPC) = 13300, *M*_w_/*M*_n_ = 1.29; *M*_n_ (NMR) = 8300] was synthesized according to a previous publication [38]. 4-(2-Hydroxyethyl)-1-piperazineethanesulfonic acid (HEPES) was ordered from Sigma-Aldrich (St. Louis, MO).

### Instruments

UV-Vis spectra were recorded with a Shimadzu UV-3600 UV-VIS-NIR spectrophotometer (Shimadzu Scientific Instruments, Columbia, MD). Fluorescence spectra were recorded with a Shimadzu RF-5301 spectrofluorophotometer. Dynamic light scattering (DLS) measurements for micelle diameters were performed using a Malvern Nano-ZS instrument equipped with a 4 mW He-Ne laser (633 nm) with an output at a scattering angle of 173°. The solution was passed through a 0.45 µm Nylon micro-filter (VWR, Batavia, IL) to remove dust before the DLS measurements. Atomic force microscopy (AFM, NanoScope III, Veeco, Plainview, NY) equipped with an integrated silicon tip/cantilever with resonance frequency of ∼240 kHz in height and phase image models was utilized for the observation of morphologies. Polymer solutions (4 µL) were dropped on a mica sample stage and dried at room temperature for morphological observation. The AFM topographies showed no evidence of tip-induced modification during successive scans.

### Preparation of micelles

Block copolymer PEG-*b*-PCL (5 mg) and 200 µL of 1 mM PtTFPP in THF were mixed to make a clear solution. The THF solution was added slowly into 1.0 mL of a MilliQ distilled water solution under vigorous stirring. The micelles were transferred to a dialysis bag and dialyzed against MilliQ water for 3 days with a water change of about every 12 hours. The solution was then filtered through a 0.45 µm micro-filter to eliminate excess non-incorporated PtTFPP. The content of PtTFPP in micelles was determined from a standard curve for PtTFPP in THF using an absorbance spectrometer. It was determined that the micelle contained 100 µM of PtTFPP, and the polymer concentration for the micelle stock solution was 2.5 mg/mL. The micelles remained stable in a 4°C refrigerator for at least one month without an alternation of photophysical properties and/or sizes.

### Determination of quantum yields

The fluorescence quantum yields (*η*) of samples in solutions were recorded by using PtTFPP in methylene chloride (*η* = 0.088) [41] excited at 390 nm and were calculated according to the following equation [42]:
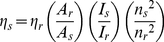
(1)where *η*_r_ and *η*_s_ are the fluorescence quantum yields of standards and the samples, respectively. *A*_r_ and *A*_s_ are the absorbance of the standards and the measured samples at the excitation wavelength, respectively. *I*_r_ and *I*_s_ are the integrated emission intensities of the standards and the samples, respectively. *n*_r_ and *n*_s_ are the refractive indices of the corresponding solvents of the solutions, respectively.

### Oxygen sensing performance

Nitrogen and oxygen gas mixtures were used to saturate the solution to adjust the dissolved oxygen concentrations. The gas mixtures were precisely controlled with a custom-built, in-line, digital gas flow controller. All sensing measurements were carried out at atmospheric pressure, 760 mmHg or 101.3 kPa. At the air saturated condition with an oxygen partial pressure of 21.4 kPa, the dissolved oxygen concentration in water is 8.58 mg/mL at 23°C.

### Response time

#### Response time measured through the bubbling with a mixture of nitrogen and oxygen gas

3 mL of PtTFPP micelles was placed in a quartz cuvette. Emission measurements were taken every 4 second at the sensor's peak emission of 650 nm and excited at 405 nm. To vary the concentrations of dissolved oxygen in the buffers, we used a gas manifold to control gas flow through a tube and needle to bubble the buffers. Holes were installed in the cuvette cap to allow the needle into the cuvette and gas flow out. Oxygen and nitrogen flow rates were set at 66 standard cubic centimeters per minute. In all trials, the buffer was first saturated with 21% of oxygen and 79% of nitrogen (to mimic atmospheric condition) before beginning the measurements. Measurements began with an immediate change from the mimic air flow into the buffer to 100% nitrogen, and then continued as the gas flow composition was then immediately reversed between air and nitrogen.

#### Response time measured using glucose oxidase

3 mL of PtTFPP micelles was placed in a quartz cuvette. Glucose was added to reach a glucose concentration of 0.25 M. 60 µL of glucose oxidase (concentration: 10 mg/mL) was added into the micelle-glucose solutions. Emission measurements were taken every 0.1 seconds at the sensor's peak emission of 650 nm and excited with 405 nm.

### Photostability investigation

The micelle solution (5 µM of PtTFPP) in pH 7.0 B-R buffer was continuously exposed to the excitation light at 405 nm (0.2 mW/cm^2^) over a period of 50 minutes. The fluorescence intensity was recorded over an interval of 2 min.

## Results and Discussion

### Micelle preparation and characterization

Using a dialysis approach as described in the experimental part, PtTFPP was successfully incorporated into the micelles. The block copolymer used for this study is a cationic amino group containing PEG-*b*-PCL (Figure 1). The use of cationic amine-containing groups can enhance the endocytosis ability, which was investigated by other research groups [43], [44] as well as by us [38].

**Figure 2:**
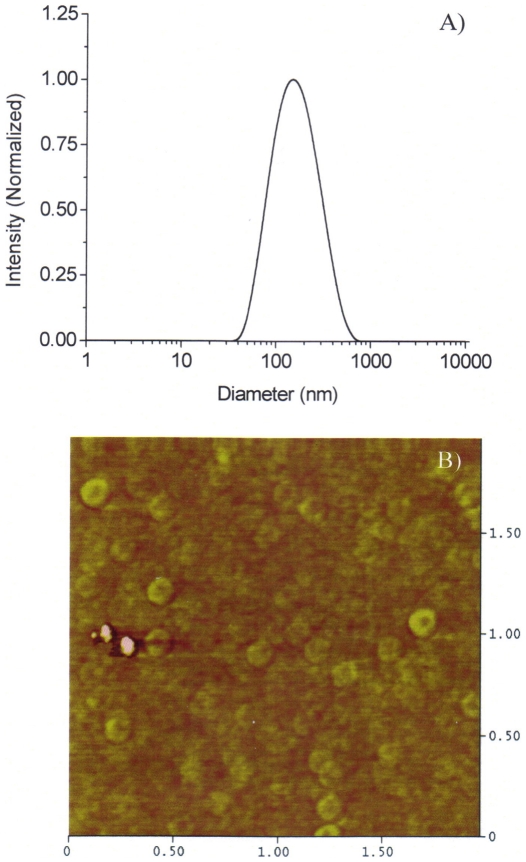
DLS of the micelles (A) and AFM image of the dried micelles (B).

Critical micelle concentration (CMC) of this PEG-*b*-PCL copolymer was determined to be 0.37 µM (3.39×10^−3^ mg/mL) [38]. Stability of the micelles formed from PEG-*b*-PCL was investigated previously, showing the micelles were stable in HEPES buffer even with 1000 fold dilution. The micelles were less stable in cell culture medium than in HEPES solution, but can still be stable in cell culture medium with 50 folds dilution [38] most likely because the medium has additional supplements such as sugars, salts, and phenol red. Micelles were characterized using DLS with an average diameter of 140 nm in the aqueous solution (Figure 2A). The micelles were dried at room temperature on mica and although the micelles easily formed a continuous phase, some micelles with an average diameter of 128±12 nm were clearly observed (Figure 2B), which is generally in accordance with the size measured in aqueous solutions.

**Figure 3:**
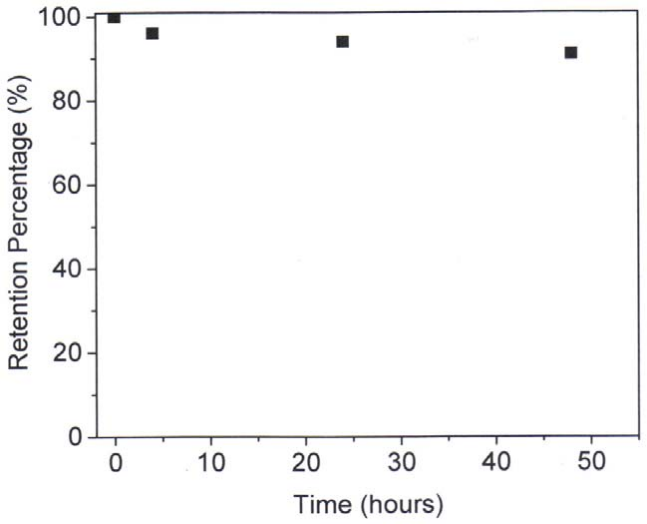
Retention percentage of PtTFPP from PCL-*b*-PEG micelles.

The micelles were quite stable when stored at 4°C. After one month storage, no size or photophysical property change was observed. Release of PtTFPP from micelles was investigated through the typical release study at 37°C in the HEPES buffer. After a 48 hours release investigation, less than 8% of PtTFPP was released from the micelles or, in other words, more than 92% of PtTFPP was retained in micelles, showing good stability of the PtTFPP incorporated PCL-*b*-PEG micelles (Figure 3).

### Photophysical properties

**Figure 4:**
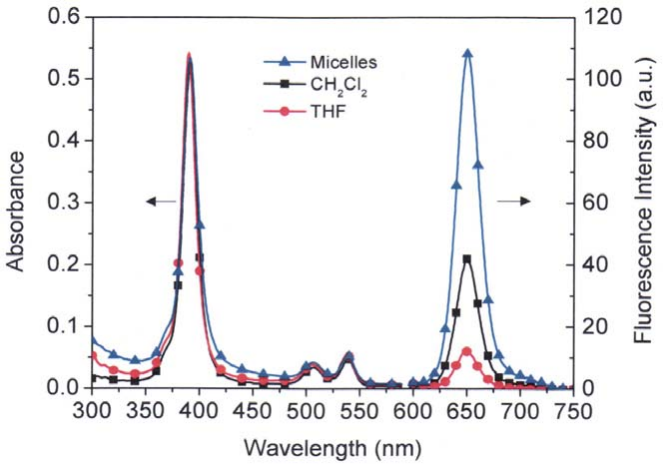
Photophysical properties of PtTFPP in micelles, THF and CH_2_Cl_2_ solutions.

Figure 4 shows the absorption and emission spectra of PtTFPP in micelles compared with those in THF and CH_2_Cl_2_ at a PtTFPP concentration of 2.5 µM. Absorbance of the micelle solution became broader than those in THF and CH_2_Cl_2_, indicating aggregations of PtTFPP molecules in the micelle cores. Interestingly, the emission of PtTFPP in the micelles was much stronger than those in THF and CH_2_Cl_2_, resulting in a higher quantum efficiency of PtTFPP in the micelles than those in THF and CH_2_Cl_2_. It was reported that quantum efficiency of PtTFPP in CH_2_Cl_2_ was 0.088 [41]. Quantum yield of PtTFPP in the micelles was calculated to be 0.110. A sugar modified PtTFPP, which is soluble in water, was reported to have a low quantum yield of 0.001 [45].

Quantum efficiency is affected by a few factors such as solvents, solvent polarity, fluorophore-fluorophore interactions, ordered and disordered aggregations [46]. Normally, the aggregated compounds show weaker emissions or exhibit lower fluorescence quantum yields than the monomeric fluorophore, due to a nonradiative decay. However, in the presence of micelles, the aggregates could be disrupted to their monomeric forms because of the distributions of fluorophores by the micellar cores formed from the hydrophobic polymers. This process will increase fluorescence quantum efficiency. When PtTFPP molecules were incorporated into the micelles, the PLC micellar cores forced the fluorophores to aggregate in the hydrophobic regions. However in the meantime, the PCL polymers could also have disrupted their aggregations. On the other hand, the polarity of PCL could also have affected the quantum yield. As a result of the combination effects of these, the quantum yield of PtTFPP in the micelles was higher than those in THF and CH_2_Cl_2_. When PtTFPP's concentrations were varied from 0.3125 µM to 2.5 µM, the quantum yields did not change much. All were within a range of 0.107 to 0.111.

### Oxygen responses of PtTFPP in micelles

**Figure 5:**
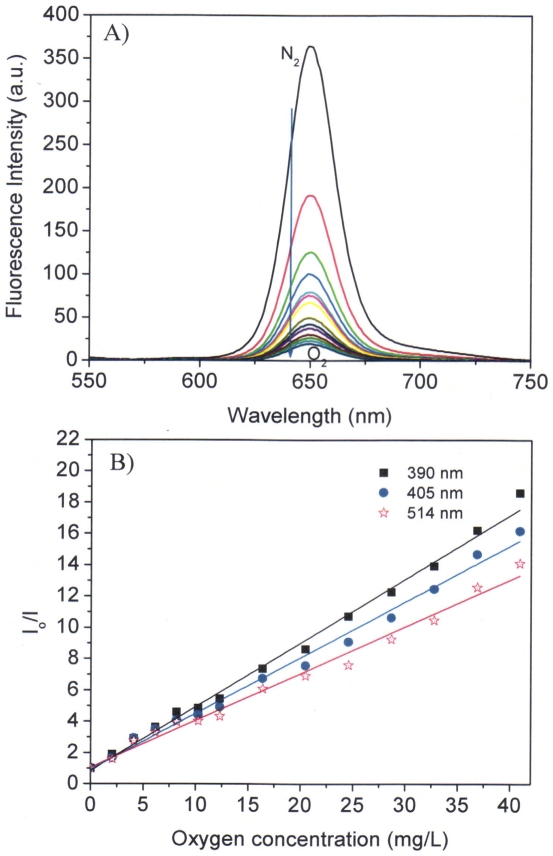
Typical oxygen sensing of the PtTFPP/PCL-*b*-PEG micelles excited at 390 nm (A). Stern-Volmer responses of the micelles excited at 390, 405, and 514 nm (B).

Dissolved oxygen concentrations were adjusted using a mixture gas of oxygen and nitrogen. The response to oxygen was shown in Figure 5A. Clearly, the emission of PtTFPP decreased with the increase of oxygen concentration. The intensity ratio (*I*_0_/*I*) curves (Figure 5B) follows the Stern-Volmer equation as given in equation 2.
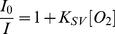
(2)where *K*_SV_ is the Stern-Volmer quenching constant. *I_0_* and *I* are the steady-state luminescence signals measured in the absence of oxygen and presence of different oxygen concentrations, respectively. [O_2_] is the dissolved oxygen concentration tuned by the nitrogen and oxygen mixed gas. Using the same optical set-up, excitation wavelength affected slightly on the Stern-Volmer quenching constants.

### Response time

**Figure 6:**
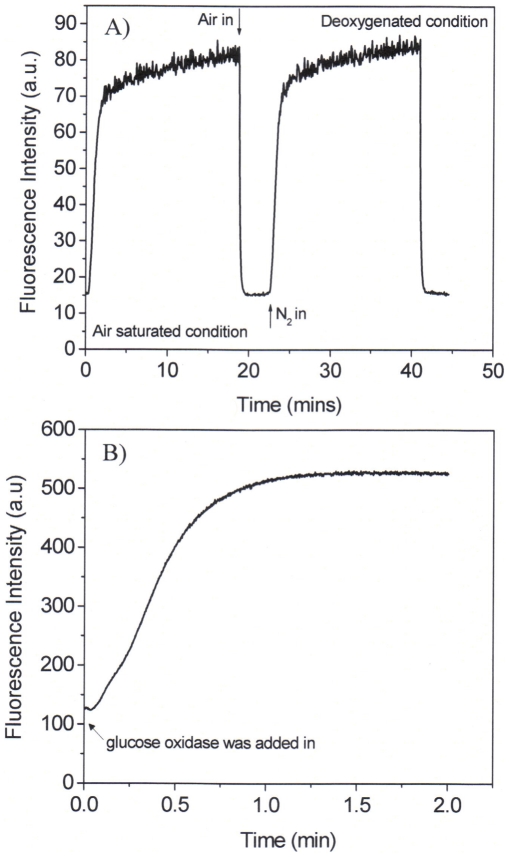
Response time studied through a saturation of air and nitrogen saturation to the micellar solutions (A) and through the consumption of the oxygen by the oxidation of glucose by glucose oxidase (B). Concentration of glucose was 0.25 M and the concentration of the glucose oxidase was 10 mg/mL.

Using the air/nitrogen saturation method, it was found the response times, *t*_95_ (i.e., the time for 95% of the total change in fluorescence intensity to occur) and *t*_99_ (i.e., the time for 99% of the total change in fluorescence intensity to occur), were 52 and 68 s from the deoxygenated condition to the air saturated condition (Figure 6A), respectively. Recovery times from the air saturated solution to deoxygenated solution were slower with a *t*_95-r_ of 380 s and *t*_99-r_ of 760 s. These response times were quite close to those of PtTFPP physically doped in poly(2-hydroxyethyl methacrylate) (PHEMA) matrix and faster than those of PtTFPP physically doped in polystyrene (PS) matrix [25]. The *t*_95_ of PtTFPP in PHEMA and PS matrices from deoxygenated solutions to oxygenated solutions are 50 and 84 seconds, respectively [25]. This comparison demonstrated that these micelles may be reasonable materials for oxygen sensing.

Stationary state oxygen responsive time was studied using the deoxygenated approach with glucose oxidase through the oxidation of glucose and consumption of oxygen. (Figure 6B) The response time *t*_95_ is 50 seconds, in accordance with the time measured using the gas saturated approach with a *t*_95_ of 52 seconds. However, this response time is much slower than that of PtTFPP incorporated in the polystyrene-*block*-polyvinylpyrrolidone beads suspended in water, which has a *t*_95_ of about 4 seconds [37]. This indicates that polystyrene-*block*-polyvinylpyrrolidone has a much faster gas permeable velocity than that of the PCL-*b*-PEG.

### Photostability of the micelles

**Figure 7:**
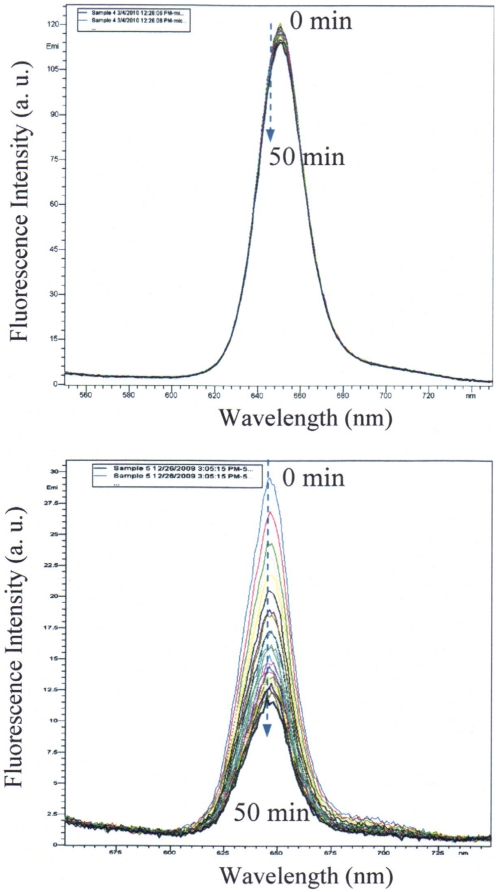
Photostability of PtTFPP in PCL-*b*-PEG micelles (A) and PtTFPP suspended in 5% THF-containing HEPES buffer (B).

Photostability of PtTFPP in the micelles was compared with that of PtTFPP suspended in water under the same experimental conditions excited at 405 nm (0.2 mW/cm^2^) for 50 minutes (Figure 7). Less than 5% fluorescence intensity decay was observed for the micelles after the 50 minutes of continuous light exposure. However, significant fluorescence decay (∼60%) of PtTFPP dispersed in water was observed. The photostability study indicated that PtTFPP in the micelles could improve the probe's photostability. There are possibly two reasons for the high photostability of PtTFPP in water: (1) The hydrophobic micelle core alleviates the direct interaction of PtTFPP with water; (2) the aggregation of PtTFPP in micelles restricts the probe's molecules' movement. As a result of these two factors, the sensors are less reactive toward photo-oxidation and reduction to increase their photostability as compared to that of PtTFPP molecules directly interacting with water.

### Conclusion

An amphiphilic block copolymer of PCL-*b*-PEG was applied to incorporate a popularly used oxygen sensor, PtTFPP, to enable the application of PtTFPP in aqueous solution. Micellar structures were observed from the PtTFPP/PCL-*b*-PEG assembled with an average diameter of 140 nm, confirmed using DLS and AFM. Interestingly, it was found that PtTFPP exhibited greater photostability in the micelles than when it was suspended in water. PtTFPP has good oxygen sensitivity and response time in the micelles, suggesting that it has great potential in its applications for oxygen sensing in biological systems. Further uses of these sensors for intracellular oxygen sensing are in progress.
